# Effect of Microstructure and Crystallographic Texture on the Fracture Toughness Anisotropy of LPBF IN718

**DOI:** 10.3390/ma18163737

**Published:** 2025-08-10

**Authors:** José David Perez-Ruiz, Wilmer Velilla-Díaz, Mikel Abasolo, Gaizka Gómez Escudero, Luis Norberto López de Lacalle

**Affiliations:** 1 School of Mechanical Engineering, Universidad Industrial de Santander, Bucaramanga 680002, Colombia; 2 Aeronautics Advanced Manufacturing Center, CFAA, 48170 Zamudio, Spain; mikel.abasolo@ehu.eus (M.A.); gaizka.gomez@ehu.eus (G.G.E.); norberto.lzlacalle@ehu.eus (L.N.L.d.L.); 3 Department of Mechanical Engineering, Escuela de Ingeniería de Bilbao, Universidad del País Vasco, 48013 Bilbao, Spain; 4 Department of Mechanical Engineering, Universidad de La Serena, La Serena 1720170, Chile; wilmer.velilla@userena.cl

**Keywords:** Inconel 718, Laser Powder Bed Fusion, additive manufacturing, fracture toughness, J-integral, build orientation, elastic–plastic fracture mechanics, crystallographic texture, anisotropy

## Abstract

Fracture toughness anisotropy is a key concern in IN718 components produced by Laser Powder Bed Fusion (LPBF), due to their strong crystallographic texture and characteristic lamellar microstructure. In this study, the effect of grain orientation on fracture toughness was evaluated by testing two LPBF IN718 builds with the same laser scanning strategy (R0), but with two different orientations: vertical (R0-0) and 45° inclined (R0-45) relative to the build direction. The mechanical response was assessed through compact tension (CT) tests following ASTM E399 and ASTM E1820 standards. Results show that the R0-45 specimens exhibited a fracture toughness nearly 2.5 times higher than R0-0 specimens. Detailed microstructural analysis, supported by EBSD and SEM, reveals that the higher toughness in the R0-45 orientation is linked to a combination of smaller effective grain size along the crack path, higher levels of geometrically necessary dislocations (GND), and increased kernel average misorientation (KAM), which collectively enhance plastic accommodation and crack-tip shielding. These findings support and reinforce the established understanding of the relationship between microstructure and anisotropic fracture behavior in LPBF IN718, facilitating its practical application in the design and orientation of additively manufactured components.

## 1. Introduction

Additive manufacturing (AM) technologies have transformed the fabrication of high-performance alloys by enabling the design of components with complex geometries and site-specific properties. Among these, Laser Powder Bed Fusion (LPBF) and Laser Directed Energy Deposition (LDED) are two widely used techniques for producing nickel-based superalloys such as Inconel 718 (IN718), known for their high strength, corrosion resistance, and fracture toughness at elevated temperatures [1,2].

The mechanical behavior of IN718 components manufactured by AM is highly dependent on processing parameters, build orientation, and post-processing procces [3,4]. Several studies have demonstrated strong agreement between experimental measurements and numerical simulations of deformation and fracture, supporting the use of advanced computational frameworks to predict mechanical behavior in both conventionally processed and additively manufactured materials [1,5,6]. These methods have revealed that the anisotropic nature and melt pool morphology strongly affect microstructural evolution, including precipitate formation and crystallographic texture [7,8]. Numerical simulations have been widely used not only to predict the mechanical behavior of metallic materials, but also to assess the impact of anisotropy on deformation and fracture processes. In anisotropic systems, such as additively manufactured alloys or architected foams, the directional dependence of mechanical properties can significantly affect stress redistribution, plastic accommodation, and crack propagation paths. To capture these phenomena, it is essential to implement anisotropic constitutive models and systematically compare their predictions against conventional isotropic approaches. González et al. demonstrated that elastic constants and material stability are strongly influenced by both structural anisotropy and defect connectivity, underscoring the need for accurate representation of microstructural features in simulation frameworks [9]. Similarly, Cruzado et al. validated fatigue crack growth predictions in LPBF Inconel 718 against experimental data using different numerical strategies, revealing that model accuracy depends strongly on microstructure-sensitive parameters such as grain orientation and residual stress state [10,11]. Building on this, recent efforts have focused on developing more advanced modeling frameworks to predict fracture processes under complex conditions. Tran et al. introduced a local–global numerical approach based on the *J*-integral, which enables accurate simulation of crack propagation considering heterogeneous microstructures and multiaxial loading [12].

Fracture resistance is a critical metric for structural applications, yet it remains challenging to assess due to the ductile nature and high energy absorption capacity of IN718. Wan et al. proposed an alternative methodology based on chip formation during milling to indirectly determine yield strength and fracture toughness, offering a practical alternative to conventional testing methods [13]. Complementary studies revealed that additive-manufactured IN718 generally exhibits superior crack initiation toughness compared to cast or wrought counterparts, due to the specific microstructural features introduced by layer-wise solidification [14]. The influence of grain boundary characteristics on fracture behavior has been further confirmed in fundamental simulations. Velilla-Díaz et al. demonstrated via molecular dynamics that high-angle grain boundaries in aluminum bicrystals significantly increase fracture toughness—by up to five times compared to single crystals—highlighting the potential of grain boundary engineering in AM alloys [15,16,17]. These insights are particularly relevant to IN718, where texture and grain misorientation can be tailored through build strategies and heat treatments [18].

Further work has examined the performance of IN718 in diverse conditions. Saju et al. investigated dissimilar metal weld joints and showed that post-weld heat treatment enhances both fracture toughness and crack growth resistance [19]. Monkova et al. quantified the effect of build orientation, reporting a 12% increase in yield strength for XZ-oriented LPBF specimens compared to those built along the Z-axis [20]. Muhammad et al. reported improved fatigue life in stress-relieved specimens due to the elimination of columnar dendritic structures [21]. Chowdhury et al. [22] have demonstrated that scanning parameters in electron beam powder bed fusion (EB-PBF) significantly influence the grain morphology and high-temperature mechanical behavior of Inconel 718. Variations in beam current, scan speed, and hatch spacing were shown to alter the temperature gradient during solidification, enabling control over the formation of columnar or equiaxed grain structures. Notably, columnar grains aligned parallel to the loading direction enhanced creep life by minimizing transverse grain boundaries, whereas equiaxed grains offered higher isotropy but reduced creep resistance. A similar microstructural sensitivity has been reported for wire-arc directed energy deposition (DED) of Inconel 718, where excessive heat input can promote segregation and coarse microstructures detrimental to mechanical performance. In particular, energy densities ranging from 360 to 540 J/mm in CMT + P-based DED were shown to increase primary dendrite arm spacing and Laves phase fraction, leading to significant reductions in as-deposited tensile strength. However, post-deposition solution-aging treatments recovered the strength to levels comparable with forged alloys, highlighting the importance of thermal control in mitigating defect formation and tailoring performance in high-deposition-rate AM processes [23]. The role of porosity and defect morphology has also been addressed. Poulin et al. found that intentional porosity significantly reduces fatigue resistance, with the effect being orientation-dependent [24]. Furthermore, advanced surface treatments such as Laser Shock Peening (LSP) were shown to delay crack propagation and enhance fracture toughness in aged IN718 alloys [25].

In summary, although AM technologies enable the fabrication of highly customized IN718 components, their fracture performance remains strongly dependent on microstructure, build orientation, and post-processing conditions. This study systematically investigates the anisotropic fracture toughness of LPBF-manufactured IN718 as a function of build orientation, providing new insights to support the optimization of process–structure–property relationships for critical structural applications.

## 2. Materials and Methods

Table 1 presents a comparison between the nominal chemical composition provided by the supplier and the experimentally measured values (in wt.%). The powder exhibited a spherical morphology and a nominal particle size range of 15–50 μm. Particle size distribution was evaluated following the ASTM B822 standard [26].

### 2.1. Materials and Specimen Preparation

**Figure 1:**
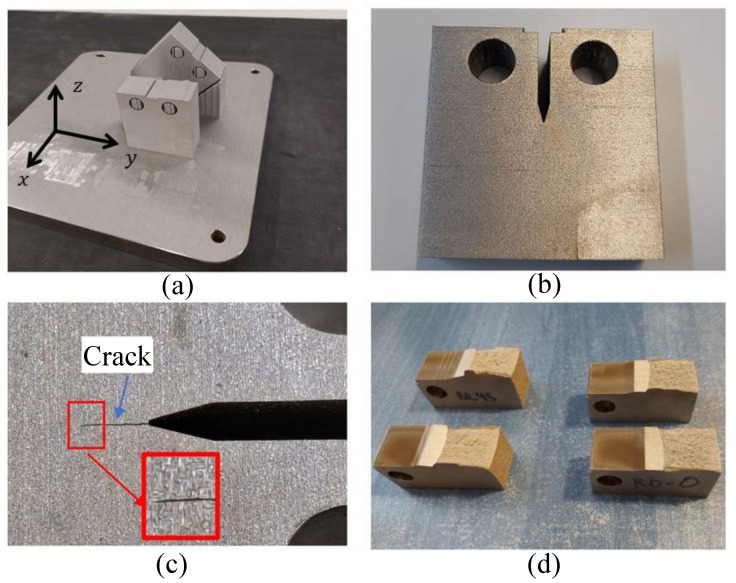
(**a**) Specimens fabricated in vertical and inclined orientations to force crack propagation along different directions relative to grain orientation and crystallographic texture. (**b**) EDM notch introduced in the specimens using EDM machine. (**c**) Fatigue pre-crack generated prior to fracture toughness testing, with a zoomed view showing the crack detail. (**d**) Overview of the fatigue pre-crack zone and final fracture surfaces, which exhibited the expected fracture behavior.

IN718 specimens were manufactured by Laser Powder Bed Fusion (LPBF) manufactured with a Renishaw AM400 (Wotton-under-Edge, UK) using a unidirectional laser scanning strategy (R0), where no rotation was applied between successive layers—ensuring a constant scan vector throughout the build. Two build orientations were selected to investigate the influence of crystallographic orientation on fracture toughness: (i) vertical orientation (R0-0), where the crack growth direction is parallel to the build direction (Z-axis), and (ii) inclined orientation (R0-45), where the crack growth direction is at 45° (see Figure 1). This approach allows direct evaluation of orientation-dependent effects on mechanical behavior and microstructural evolution. Table 2 presents the manufacturing parameters. The processing parameters employed enable the attainment of a high relative density (ρ > 99.9%) [18].

Fracture toughness testing was performed using compact tension (CT) specimens, prepared according to ASTM E399 [27] and ASTM E1820 standards [28]. The manufactured specimens promote the development of a plane stress state, which is recommended for the type of analysis conducted in this study. Additionally, it is important to note that the microstructure produced by LPBF using the R0 strategy exhibits both microstructural and crystallographic patterns that are consistently formed throughout the material [18]. As a result, although the sample is macroscale in size, these microstructural patterns exert a macroscopic influence across the entire specimen. The testing configuration, specimen dimensions, and mechanical parameters are summarized in Table 3.

Microstructural characterization was performed using scanning electron microscopy (SEM) and electron backscatter diffraction (EBSD). SEM and EBSD analyses were performed with a Sigma 500 Zeiss FESEM (Oberkochem, Germany) and an Oxford C-NANO detector. Three main aspects were analyzed to understand the relationship between microstructure and fracture resistance: grain morphology, Kernel Average Misorientation (KAM), and geometrically necessary dislocation (GND) density. Grain morphology was quantified by modeling individual grains as ellipsoids with three principal axes. For each specimen orientation, the effective grain length was calculated as the intersection between the fracture plane and the modeled ellipsoid geometry, as illustrated in Section 3.4. This provided a relevant measure of the microstructural scale influencing crack propagation.

**Figure 2:**
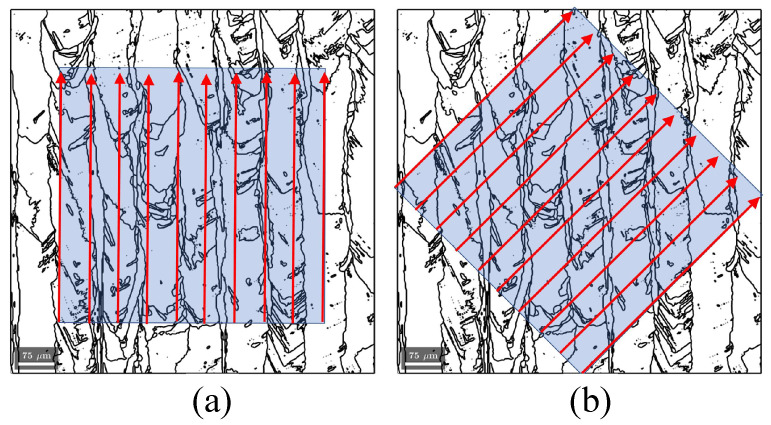
Methodology used for extracting KAM and GND profiles along the grain structure. Ten analysis lines were positioned within an area of 550×550μm: (**a**) vertical lines (0°) in a horizontal frame for R0-0 specimens; (**b**) diagonal lines (45°) in a rotated frame for R0-45 specimens.

KAM and GND analyses were conducted on EBSD maps to evaluate lattice curvature and local dislocation density. To capture orientation effects, profiles of KAM and GND were extracted along ten vertical and diagonal (45°) lines traversing the grain structure. The lines were selected within an analysis area of 550×550μm, using a rectangular horizontal frame for the R0-0 specimens and a rotated diagonal frame for the R0-45 specimen, as illustrated in Figure 2. This approach enabled a direct comparison of plastic accommodation and dislocation structures as a function of specimen orientation and crack path, providing further insight into the role of microstructure in fracture behavior. The black lines shown in Figure 2 represent the grain boundaries of the sample as revealed by EBSD analysis.

To interpret the experimental EBSD results within a physically meaningful framework, we refer to the crystal plasticity concepts introduced by Cruzado et al. [10,11], which provide a solid basis for understanding the elasto-viscoplastic behavior of IN718 at the grain scale. In this context, the crystals are considered as elasto-viscoplastic solids, and the plastic slip rate and kinematic hardening on each slip system are conceptually described by Equations (1)–(4). Although no computational simulations are performed in this study, these equations serve to support the interpretation of the deformation mechanisms inferred from the EBSD data, particularly those related to dislocation activity and crystallographic anisotropy.

(1) $${\dot{\gamma }}^{\alpha }={\dot{\gamma }}_{0}{\left(\frac{|\tau^{\alpha }-\chi^{\alpha }|}{g^{\alpha }}\right)}^{1/m}\mathrm{sign}\left(\tau^{\alpha }-\chi^{\alpha }\right)$$

(2) $$\chi^{\alpha }=c\;{\dot{\gamma }}^{\alpha }-d\;\chi^{\alpha }\left|{\dot{\gamma }}^{\alpha }\right|{\left(\frac{|\chi^{\alpha }|}{c/d}\right)}^{mk}$$

(3) $$c=c^{\prime }+\frac{K_{c}}{\sqrt{D}}$$

(4) $$\frac{c}{d}={\left(\frac{c}{d}\right)}^{\prime }+\frac{K_{c/d}}{\sqrt{D}}$$

Here, ${\dot{\gamma }}^{\alpha }$ represents the plastic slip rate for slip system α; ${\dot{\gamma }}_{0}$ is the reference strain rate; *m* is the rate sensitivity parameter; $g^{\alpha }$ is the critical resolved shear stress (CRSS); $\chi^{\alpha }$ is the back-stress or kinematic hardening; and mk is a parameter controlling the mean stress relaxation velocity. The evolution of the kinematic hardening is governed by the hardening modulus *c*, while the ratio c/d defines the saturation value of the kinematic hardening. Additionally, the grain size dependence of these parameters is introduced through the Hall–Petch terms $K_{c}$ and $K_{c/d}$, which relate to the effective grain size *D*, thus allowing the model to incorporate grain size effects on both the initial and saturation hardening behavior.

It is essential to emphasize that the evolution of plastic slip rate and kinematic hardening is inherently interdependent. According to Equations (1)–(4), the plastic slip rate ${\dot{\gamma }}^{\alpha }$ is governed by the effective resolved shear stress, defined as the difference between the applied shear stress and the back stress ($\tau^{\alpha }-\chi^{\alpha }$). As $\chi^{\alpha }$ increases, this driving force diminishes, thereby suppressing further slip activity. Simultaneously, the back stress $\chi^{\alpha }$ evolves as a function of the accumulated slip, as delineated in Equation (2), establishing a dynamic feedback loop. This coupling creates a self-regulating mechanism wherein progressive plastic deformation enhances kinematic hardening, which in turn stabilizes the plastic zone by limiting additional slip.

By combining experimental EBSD data with physically based crystal plasticity concepts, the methodology enables a mechanistic interpretation of how build orientation influences local deformation behavior and, by extension, fracture toughness in LPBF-fabricated IN718. It is worth noting that, in the as-built condition, the microstructure primarily consists of matrix and Laves phases [29], with the latter forming predominantly in interdendritic regions. Given its relatively uniform distribution and localized nature, the Laves phase is not expected to significantly affect the observed anisotropy in fracture toughness.

### 2.2. Fracture Toughness Calculation via J-Integral

Fracture toughness was evaluated using two complementary approaches based on the *J*-integral framework: Method 1 (M1), which strictly follows the standardized procedure outlined in ASTM E1820 for compact tension specimens, and Method 2 (M2), a simplified estimation derived from a reduced form of Rice’s path-independent integral. While M1 ensures normative accuracy, M2 is intended as a semi-analytical approximation that facilitates rapid assessment using minimal input data. Although this method introduces notable simplifications and is not standards-compliant, it is included to explore its potential to qualitatively capture orientation-dependent differences in fracture behavior. This comparative perspective aims to highlight the methodological sensitivity of toughness estimations and may offer practical value in data-limited modeling scenarios or preliminary evaluations.

#### 2.2.1. Standardized Method (M1): ASTM E1820 for CT Specimens

In this approach, the total *J*-integral is calculated as the sum of its elastic and plastic components:

(5) $$J=J_{\mathrm{el}}+J_{\mathrm{pl}}$$

The elastic component $J_{\mathrm{el}}$ is calculated using the stress intensity factor *K* as:

(6) $$J_{\mathrm{el}}=\frac{K^{2}}{E^{\prime }}$$

The plastic component $J_{\mathrm{pl}}$ is determined from the plastic work done during loading. This is calculated using numerical integration of the area under the load-displacement curve:

(7) $$J_{\mathrm{pl}}=\frac{\eta A_{pl}}{B_{N}b_{o}}$$

where $A_{pl}$ is the plastic area obtained from the experimental load-displacement data, $B_{N}$ is the net specimen thickness, $b_{o}$ is the uncracked ligament (W−a), and $\eta =2+0.522\;\cdot \;b_{o}/W$ is a geometry-dependent factor. The final value of *J* is used to estimate the fracture toughness $J_{\mathrm{IC}}$, following the guidelines of ASTM E1820.

#### 2.2.2. *J*-Integral Estimation via Simplified Contour Integration (M2): Rice’s Path-Independent Integral

**Figure 3:**
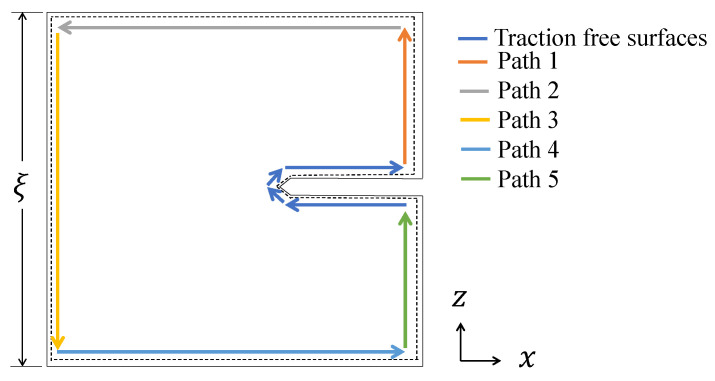
Integration path for the *J*-integral.

The fracture toughness was estimated from the calculated *J*-integral as a path-independent line integral around the crack tip proposed by Rice [30]:

(8) $$J=\int_{\Gamma }\left(wdz-T_{i}\frac{\partial u_{i}}{\partial x}\right)\;ds$$

where *J* is the *J*-integral, representing the energy release rate per unit crack extension; Γ is a contour surrounding the crack tip along which the integration is performed (Γ = Path 1 + Path 2 + Path 3 + Path 4 + Path 5 + Traction free surfaces in Figure 3); *w* is the strain energy density, which is calculated from the stress tensor $\sigma_{ij}$ and strain $\varepsilon_{ij}$ as follows:

(9) $$w=\int_{0}^{\varepsilon_{ij}}\sigma_{ij}\;d\varepsilon_{ij}$$

$T_{i}$ is the *i*-th component of the traction vector, given by

(10) $$T_{i}=\sigma_{ij}n_{j}$$

Here, $n_{j}$ is the outward unit normal to the contour Γ; $u_{i}$ is the *i*-th component of the displacement vector; and ds is the length increment along the contour Γ.

This evaluation incorporated material-specific considerations and assumed a representative contour path to approximate the near-tip energy release:

1. Along the segments corresponding to the traction free surfaces, it holds that $T_{i}=0$ and dz=0. Thus, by referring to Figure 3, the total contour integral can be decomposed as:
  (11) $$J=J_{P1}+J_{P2}+J_{P3}+J_{P4}+J_{P5}$$
2. For the outer segments, Path 1 and Path 5, direct evaluation of $\sigma_{ij}$ confirms that both the tractions and the strain energy density vanish. As a result, $T_{i}=0$, w=0, and consequently $J_{P1}=J_{P5}=0$.
3. For the crack surfaces, Path 2 and Path 4, the condition dz=0 remains valid. Additionally, the traction vectors take the form $T_{i}=\left(0,0,\sigma_{ij}\right)$, and the displacement gradients along *x* are given by $\partial u_{i}/\partial x=\left(\partial u_{x}/\partial x,0,0\right)$; therefore $T_{i}\;\cdot \;\partial u_{i}/\partial x=0$, yielding $J_{P2}=J_{P4}=0$.
4. Finally, due to the traction-free boundary condition on Path 3, the normal traction vanishes, i.e., $T_{i}=0$.

From the above, the only non-zero contribution comes from Path 3, implying $J=J_{P3}$. Therefore, the expression for the *J*-integral becomes:

(12) $$J=\int_{\mathrm{Path}\;3}w\;dz=\xi \;w_{\mathrm{Path}\;3}=\xi \int_{0}^{\varepsilon_{zz}}\sigma_{zz}\;\varepsilon_{zz}$$

where ξ is the total height of specimen after fracture, as illustrated in Figure 3. The strain energy is calculated considering an equivalent strain in function of the crack opening displacement (COD): $\varepsilon =COD/L_{o}$ and equivalent stress component as follows:

(13) $$\sigma_{zz}=\frac{P}{b_{o}\;\cdot \;B}$$

In this formulation, only the $\sigma_{zz}$ component from Path 3 is retained, while the transverse stress components are neglected for simplification. The results from both methods are presented and compared in the following sections.

## 3. Results

**Figure 4:**
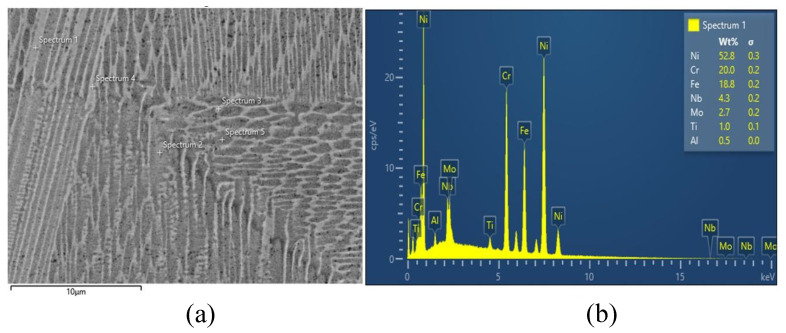
EDS analysis: (**a**) SEM micrograph showing the analyzed regions for EDS measurements; (**b**) EDS spectrum corresponding to Spectrum 1, indicating the chemical composition of the LPBF-manufactured IN718 sample.

An EDS analysis was performed on the fabricated sample to determine the chemical composition of IN718 after the LPBF process. The results are shown in Figure 4 and display values similar to those reported in the Table 1.

### 3.1. Elastic Components: Stress Intensity Factor and J-Integral

The fracture toughness, expressed in terms of the *J*-integral, was experimentally determined for both build orientations: R0-0 and R0-45. The evaluation followed the procedures outlined in ASTM E1820, using the load–displacement data and the geometric parameters summarized in Table 3. The corresponding stress intensity factors *K* were computed for each condition based on the applied load, specimen geometry, and material properties listed in Table 3. The dimensionless geometric function f(a/W), as defined by ASTM E1820, was employed to account for the specific crack-to-width ratio in each specimen. These *K* values were subsequently used to calculate the elastic component of the *J*-integral, $J_{\mathrm{el}}$, through Equation (6). The results for each orientation are summarized in Table 4.

### 3.2. Fracture Toughness ($J_{\mathrm{IC}}$)

**Figure 5:**
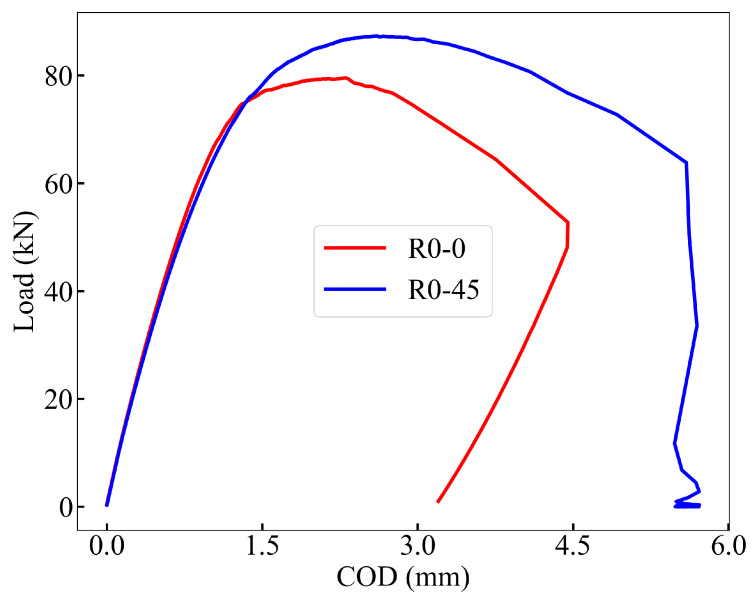
Load vs. COD curves for specimens R0-0 and R0-45.

For the first method (M1), the plastic work area ($A_{pl}$) was computed by numerically integrating the load vs. COD curve (see Figure 5) using Simpson’s 1/3 rule. Subsequently, the plastic component of the *J*-integral, $J_{\mathrm{pl}}$, was estimated using Equation (7), and the total fracture toughness $J_{\mathrm{IC}}$ was calculated as the sum of its elastic and plastic contributions, following Equation (5). In the second method (M2), the equivalent stress and strain were computed from the load vs. COD curve and ξ was obtained from the fracture specimens. The results are summarized in Table 5.

Although the fracture toughness values estimated by the two methods differ by approximately 20 percent, primarily due to the simplifications in M2, the relative trend remains consistent. Both approaches indicate a significant improvement in fracture toughness for the R0-45 orientation compared to R0-0, with an increase approaching a factor of three.

### 3.3. Fractographic Analysis Based on SEM Observations

**Figure 6:**
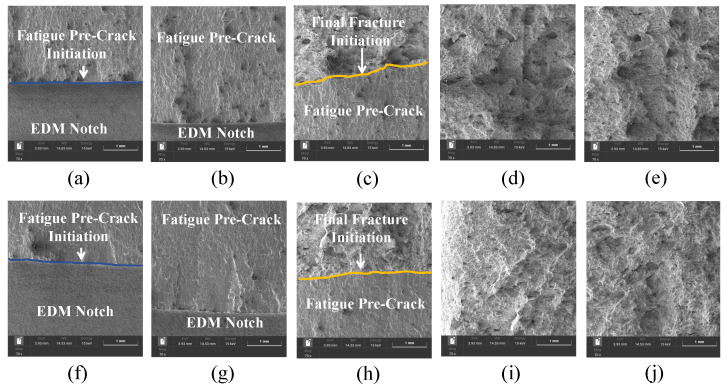
Fracture surface features of LPBF-IN718 CT specimens captured by SEM. Top row (**a**–**e**): R0-0 orientation showing (**a**) interfacial fatigue pre-crack initiation, (**b**,**c**) early and mid-stage fatigue pre-crack propagation, (**d**) onset of final fracture, and (**e**) quasi-cleavage final fracture. Bottom row (**f**–**j**): R0-45 orientation showing (**f**) fatigue pre-crack initiation in well-fused zone, (**g**,**h**) transition from fatigue to final fracture with ductile tearing, and (**i**,**j**) ductile overload fracture via dimple rupture.

To correlate fracture toughness with failure micromechanisms, fractographic analysis was conducted using SEM on CT specimens built in R0-0 and R0-45 orientations, as shown in Figure 6.

R0-0 orientation: Figure 6a shows the fatigue pre-crack initiation at interlayer boundaries adjacent to the EDM notch, characterized by intergranular decohesion and lack-of-fusion features indicative of weak metallurgical bonding. In Figure 6b,c, the fatigue pre-crack propagates along planar facets with poorly defined striations, suggesting limited plastic deformation during cyclic loading. Figure 6d reveals a transition zone with isolated microvoids and shallow dimples, while the final fracture surface in Figure 6e is dominated by quasi-cleavage and transgranular facets, which are hallmarks of a brittle fracture mode associated with low fracture toughness.

R0-45 orientation: In contrast, Figure 6f depicts pre-crack initiation in a defect-free region with homogeneous melt pool consolidation. The fatigue propagation regions shown in Figure 6g,h exhibit well-developed striations and extensive microvoid coalescence, indicating stable crack growth with significant plastic accommodation. Figure 6i,j correspond to the transition and final fracture zones, respectively, and are characterized by ductile tearing and large equiaxed dimples with shear lip formation. These features are consistent with a ductile rupture mechanism and correlate with the elevated $J_{\mathrm{IC}}$ values measured for this orientation. These observations support the mechanical results: R0-0 specimens fail via premature brittle fracture due to poor interlayer bonding, while R0-45 specimens exhibit ductile tearing governed by enhanced plastic accommodation and energy dissipation mechanisms.

These observations support the mechanical results: R0-0 specimens fail via premature brittle fracture due to poor interlayer bonding, while R0-45 specimens exhibit ductile tearing governed by enhanced plastic accommodation and energy dissipation mechanisms.

### 3.4. Orientation-Dependent Fracture Behavior

**Figure 7:**
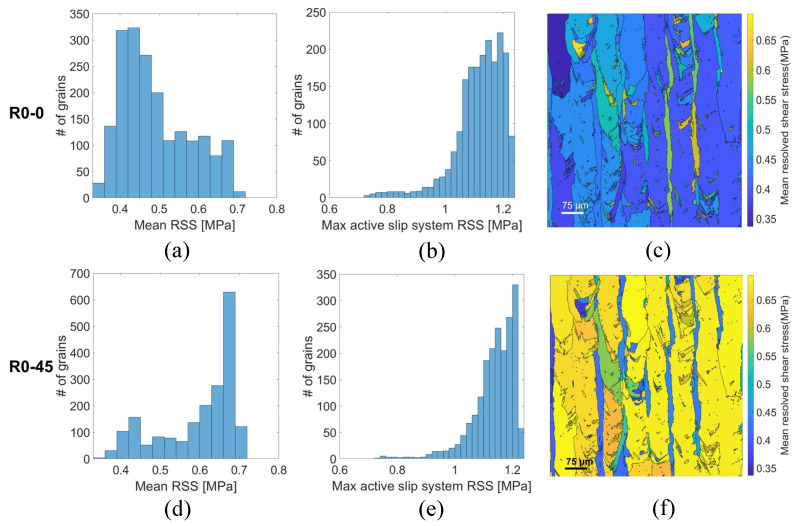
Distribution of resolved shear stress (RSS) in R0-0 (top row) and R0-45 (bottom row) specimens. (**a**,**d**) Mean RSS values calculated across all twelve FCC slip systems per grain. (**b**,**e**) Maximum RSS value for the most active slip system per grain. (**c**,**f**) Spatial maps of mean RSS per grain obtained from EBSD data. Higher RSS values in R0-45 specimens indicate greater plastic slip activity and contribute to the improved fracture toughness observed in this orientation.

Section 3.3 reveals that the superior fracture toughness observed in R0-45 specimens cannot be attributed solely to macroscopic orientation effects. Instead, it arises from microstructural mechanisms such as crack tip blunting, deflection, and increased crack path tortuosity, all of which contribute to enhanced energy dissipation and damage tolerance. To elucidate the microstructural origins of this anisotropic fracture response, it is necessary to examine the evolution of crystallographic orientation, slip activity, and hardening behavior within the material. Figure 7 presents a comparative analysis of the resolved shear stress (RSS) distributions and corresponding spatial maps for R0-0 (top row) and R0-45 (bottom row) specimens. The histograms quantify two critical parameters: (a,d) the average RSS across all twelve FCC slip systems for each grain, and (b,e) the peak RSS associated with the most highly stressed slip system. Complementary maps (c,f) illustrate the grain-level spatial distribution of the average RSS, highlighting differences in local stress heterogeneity and slip system activation between orientations.

**Figure 8:**
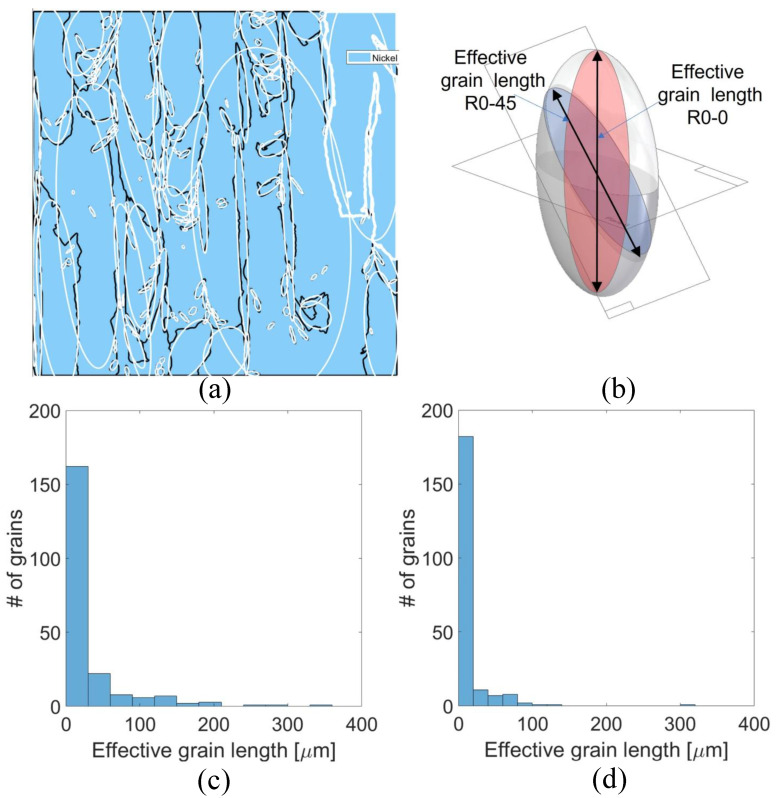
Effective grain length analysis for R0-0 and R0-45 orientations. (**a**) ellipsoid method to fit grain dimensions. (**b**) Schematic illustrating the determination of effective grain length along different crack propagation planes. (**c**) Distribution of effective grain length for R0-0 specimens. (**d**) Distribution of effective grain length for R0-45 specimens.

Figure 8 illustrates the influence of grain morphology and effective grain size on fracture resistance as a function of build orientation. Given the highly columnar grain structure produced by the LPBF process, the effective grain size experienced by a propagating crack is highly dependent on its orientation relative to the build direction and the principal grain axes. In this study, individual grains were approximated as ellipsoids defined by three orthogonal principal axes, enabling an orientation-sensitive estimation of the microstructural barrier presented to crack propagation. In the R0-0 configuration, the fatigue pre-crack propagated parallel to the build direction (Z-axis), effectively aligning with the major axis of the elongated columnar grains—thus minimizing the frequency of grain boundary interactions along the crack path.

**Figure 9:**
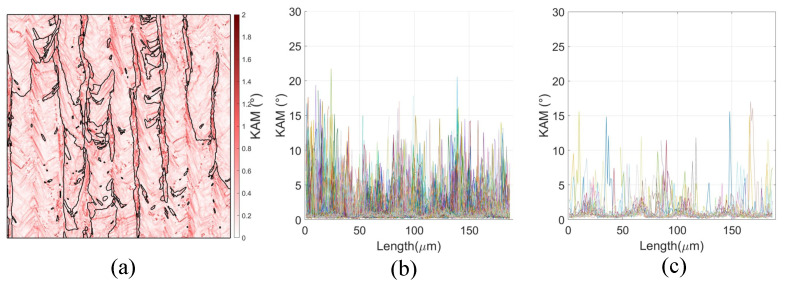
Kernel Average Misorientation (KAM) analysis for LPBF IN718 specimens. (**a**) KAM map highlighting local lattice curvature and strain distribution. (**b**) KAM profiles extracted along 45° lines (R0-45 orientation). (**c**) KAM profiles extracted along vertical lines (R0-0 orientation). The higher KAM levels in R0-45 specimens reflect increased local misorientation and plastic accommodation, contributing to enhanced fracture toughness.

Figure 9 presents the distribution of Kernel Average Misorientation (KAM) for the R0-45 specimen, offering insights into local lattice curvature and the corresponding density of geometrically necessary dislocations (GNDs). The KAM map (Figure 9a) reveals elevated misorientation levels in grains exhibiting <001> crystallographic alignment, particularly concentrated along grain boundaries and interdendritic regions. These localized increases in KAM suggest enhanced plastic strain gradients and a higher accumulation of GNDs in areas of crystallographic incompatibility, which may contribute to the observed resistance to crack propagation through mechanisms such as strain hardening and crack-tip shielding. This is consistent with prior observations in LPBF-processed IN718, where heterogeneous thermal gradients and solidification-induced residual stresses promote lattice distortions at grain interfaces. Figure 9b,c further quantify the variation in KAM along defined linear paths.

**Figure 10:**
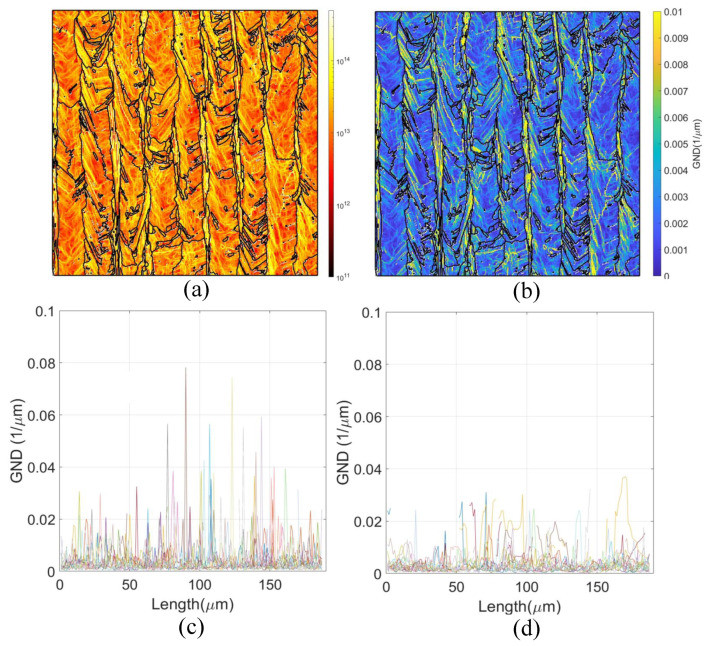
Geometrically Necessary Dislocation (GND) analysis in LPBF IN718 specimens. (**a**) Dislocation energy map derived from EBSD data. (**b**) GND density map showing local variations in dislocation content. (**c**) GND profiles extracted along 45° lines (R0-45 orientation). (**d**) GND profiles extracted along vertical lines (R0-0 orientation). Higher GND levels along the 45° paths indicate enhanced plastic accommodation in R0-45 specimens, consistent with their higher fracture toughness.

Figure 10 offers a detailed view of the spatial distribution of geometrically necessary dislocations (GNDs) and their contribution to fracture resistance in the R0-45 specimen. The dislocation energy map (Figure 10a) and the corresponding GND density distribution (Figure 10b) highlight regions of intensified dislocation activity. GND profiles extracted along diagonal (45°) and vertical (0°) lines—shown in Figure 10c and Figure 10d, respectively—further elucidate the orientation-dependent nature of plastic accommodation. Notably, the GND distribution mirrors the patterns identified in the KAM analysis: regions exhibiting elevated KAM also display increased GND density, indicating a strong correlation between local lattice curvature and dislocation content. This alignment underscores the role of microstructural anisotropy in governing the plastic response ahead of the crack tip.

## 4. Discussion

This study demonstrates a clear anisotropy in the fracture toughness of LPBF-fabricated IN718, strongly influenced by build orientation. Specimens produced in the R0-45 orientation exhibited significantly higher $J_{\mathrm{IC}}$ values—approximately 2.5 to 3 times greater—than those built in the R0-0 configuration. While this difference aligns with previous observations on the directional dependence of mechanical performance in LPBF materials, our results provide further insight into the underlying micromechanisms that govern this behavior.

The dominant contribution of the plastic component to the total *J*-integral, observed in both orientations, confirms that fracture proceeds under ductile conditions at room temperature. However, the R0-0 specimens showed early crack initiation and abrupt failure, as evidenced by quasi-cleavage features, intergranular decohesion, and lack of fusion between melt layers observed in SEM fractography. These features reveal a limited capacity for plastic accommodation along the crack front, likely associated with unfavorable grain boundary alignment and anisotropic residual stress accumulation.

In contrast, the R0-45 specimens exhibited a markedly different fracture mode. SEM images revealed stable crack propagation zones with well-defined fatigue striations and widespread microvoid coalescence, indicating enhanced plasticity and delayed failure. This morphological evidence suggests that crack-tip blunting, deflection across heterogeneous grain orientations, and a more tortuous crack path all contribute to improved energy dissipation.

These observations were reinforced by EBSD-based microstructural characterization. The R0-45 specimens presented elevated KAM and GND densities along the crack propagation direction, reflecting high levels of localized plastic strain. Such features are indicative of active dislocation mechanisms near the crack tip, which provide a crack-tip shielding effect by redistributing stresses and impeding crack advance. The presence of these dislocation structures, coupled with finer effective grain sizes intersected along the inclined crack path, results in a tougher and more damage-tolerant response.

A marked increase in RSS is observed for the R0-45 specimens (See Figure 7). Specifically, both the mean RSS and the maximum RSS show significantly higher values in R0-45, as evidenced by the shift in histogram peaks toward larger stresses and by the greater prevalence of yellow regions in the grain-resolved RSS map. In contrast, R0-0 specimens exhibit lower RSS distributions with dominant blue regions, indicating reduced resolved shear stress magnitudes. These differences can be interpreted in light of crystallographic orientation effects on slip activity. Higher RSS levels across grains and slip systems imply that a larger fraction of the crystal lattice is favorably oriented to accommodate plastic deformation under the applied loading direction. In turn, this facilitates enhanced plastic slip rates—since, according to crystal plasticity theory, slip rate increases exponentially with RSS relative to the critical resolved shear stress (CRSS) for each slip system. Consequently, the R0-45 orientation promotes more widespread activation of slip systems and greater dislocation motion. This effect contributes directly to fracture toughness. Higher plastic slip rates enable more efficient energy dissipation at the crack tip through mechanisms such as dislocation pile-ups, crack tip blunting, and microvoid nucleation. Moreover, the enhanced plasticity promotes a larger and more stable plastic zone, delaying crack propagation and increasing the energy required for fracture, as reflected in the elevated $J_{\mathrm{IC}}$ values for R0-45. It is also noteworthy that the elevated RSS and slip activity in R0-45 specimens complement the microstructural factors discussed earlier—namely, the reduction in effective grain size along the crack path and the associated increase in GND density and KAM. Together, these factors produce a microstructure that simultaneously offers higher strength (via Hall–Petch and GND hardening) and greater ductility (via active slip and plastic accommodation), both of which are critical contributors to enhanced fracture toughness.

Indeed the grain morphology is a significant aspect. Because of it induce a larger effective grain size along the crack path for the R0-0 orientation, as visualized in the schematic of Figure 8b and quantified in the distribution shown in Figure 8c. In contrast, for the R0-45 orientation, the crack path intersects the grains at an oblique angle (45° relative to Z), corresponding to a shorter effective grain length, as shown in Figure 8d. The average effective grain size is approximately 33 μm for R0-0 and 15 μm for R0-45 specimens. This reduction in effective grain size for R0-45 increases the resistance to crack propagation via the well-established Hall–Petch mechanism, wherein grain boundaries act as obstacles to dislocation motion and crack advance. A smaller effective grain size along the crack path introduces more frequent barriers to plastic flow and enhances local strain hardening, thereby elevating the fracture toughness component associated with material strength. Figure 8a further corroborates these observations, showing the orientation of the grain ellipsoids predominantly aligned along the build direction. The schematic in Figure 8b emphasizes the geometric relationship between crack path orientation and effective grain length, underscoring how the R0-45 condition inherently promotes a microstructure with higher resistance to crack growth. Taken together, these results highlight that the observed improvements in fracture toughness for R0-45 specimens are not solely a function of slip activity (as indicated by RSS maps), but also stem from microstructural refinement in the effective grain size along the fracture plane. This dual contribution—higher Hall–Petch strengthening and enhanced slip-mediated ductility—provides a robust explanation for the superior fracture performance of the R0-45 orientation. Following the methodology described earlier (diagonal and vertical lines across the sample ), it is evident that KAM values measured along diagonal paths at 45° reach significantly higher levels—frequently exceeding 10°—compared to those along vertical paths, where KAM remains much lower. This reflects two key microstructural effects: (i) the change in crystallographic orientation between adjacent grains is more pronounced along oblique paths, forcing the measurement line to intersect multiple grain boundaries and substructure domains; (ii) the dendritic substructure in LPBF IN718 exhibits a preferential diagonal alignment, further elevating lattice curvature and GND density along such orientations. From a mechanical standpoint, higher KAM values correlate with increased local GND density, which contributes to back-stress hardening in the plastic zone surrounding the crack tip. This hardening mechanism not only enhances the effective yield strength locally, but also promotes greater strain hardening capacity—both of which are essential for improving fracture toughness in ductile materials. Elevated KAM along the 45° orientation path therefore provides additional resistance to crack propagation by increasing the energy required for crack advance and promoting a more stable crack growth regime. In combination with the previously discussed effects of reduced effective grain size and increased resolved shear stress in the R0-45 configuration, the elevated KAM and GND density act synergistically to further enhance the toughness of these specimens. The presence of higher KAM reflects a microstructural state with superior capacity for plastic accommodation, greater crack-tip blunting, and improved damage tolerance—all of which are critical for achieving the elevated $J_{\mathrm{IC}}$ values observed experimentally.

Regarding the GND, it is interesting to observe that along diagonal paths (Figure 10c), GND peaks often reach values as high as 0.08 μm^−1^, while along vertical paths (Figure 10d), GND values rarely exceed 0.04 μm^−1^. This confirms that crack paths aligned at 45° intersect zones of higher lattice curvature and higher GND content, which are largely driven by grain boundary misorientations and the alignment of the underlying dendritic structure. From a fracture mechanics perspective, elevated GND density has a well-recognized influence on crack resistance. Local accumulations of GNDs contribute to back-stress hardening by generating internal stress fields that oppose dislocation motion. Each peak in GND density represents a microstructural barrier that can impede crack advance. As a result, the presence of such barriers increases the energy required for crack propagation, contributing to the overall fracture toughness of the material. Moreover, the observed synergy between KAM and GND maps indicates that the R0-45 orientation not only promotes greater slip activity (as evidenced by RSS and plastic slip rates) but also enhances the formation of dislocation structures capable of resisting further deformation. This dual effect supports stable crack growth by increasing both the strength and ductility of the crack-tip plastic zone. Importantly, the fact that GND peaks are more prominent along the 45° paths aligns with the experimental finding that R0-45 specimens exhibit higher $J_{\mathrm{IC}}$ values. The accumulation of GNDs in these orientations leads to more effective crack-tip shielding, delaying crack extension and enabling greater energy dissipation prior to failure The superior fracture toughness exhibited by the R0-45 specimens results from a synergistic combination of microstructural and mechanical factors. The inclined crack path effectively reduces the encountered grain size, increasing crack propagation resistance through Hall–Petch strengthening. Simultaneously, elevated GND densities and enhanced KAM values reflect a microstructure capable of accommodating greater plastic deformation, contributing to local hardening and improving crack tip stability. Moreover, higher resolved shear stresses promote increased slip activity across multiple crystallographic systems, enabling more extensive dislocation motion, crack tip blunting, and energy dissipation. Together, these mechanisms facilitate a more ductile fracture process in R0-45 specimens, as confirmed by SEM fractography, and underpin their markedly higher $J_{\mathrm{IC}}$ values. These findings emphasize the importance of build orientation as a key parameter for tailoring the damage tolerance of LPBF-fabricated IN718 components through microstructure-informed design strategies.

Overall, the enhanced fracture resistance in the R0-45 orientation cannot be attributed solely to macroscopic build direction. Rather, it arises from a synergistic interaction between crystallographic texture, grain boundary misorientation, slip system activity, and microstructural constraints that govern plasticity. These findings emphasize the critical role of orientation-sensitive micromechanical mechanisms in the fracture behavior of LPBF IN718 and highlight the importance of integrating microstructure-informed design strategies for critical structural applications.

## 5. Conclusions

This study assessed the fracture toughness of Inconel 718 specimens produced by Laser Powder Bed Fusion (LPBF), focusing on the effect of build orientation. Based on the experimental results and analysis performed, the following conclusions are drawn:

- Build orientation significantly influences the fracture resistance of LPBF-fabricated IN718. Specimens oriented at R0-45 demonstrated nearly 2.5 times higher $J_{\mathrm{IC}}$ values compared to those built at R0-0, indicating superior crack growth resistance.
- The fracture behavior was governed by elastic–plastic deformation, with the plastic component contributing substantially to the total *J*-integral in both orientations. This confirms the relevance of using elastic–plastic fracture mechanics criteria in AM nickel-based superalloys.
- The adopted methodology, combining compact tension specimens with compliance-based crack length estimation and ASTM E1820 procedures, proved robust for characterizing fracture toughness in LPBF components.
- The findings highlight the importance of build orientation as a critical parameter in the design and qualification of AM parts. The R0-45 orientation offers enhanced damage tolerance and should be prioritized in structural applications.
- These insights provide a foundation for future studies aiming to link microstructure and fracture behavior more explicitly, and to optimize additive manufacturing parameters for high-performance applications.
- The superior fracture toughness observed in the R0-45 sample is closely linked to microstructural and crystallographic features. Enhanced plastic deformation capacity, increased GND density, elevated KAM values, and higher resolved shear stress levels collectively promote crack-tip shielding and energy dissipation. Furthermore, the oblique crack path reduces the effective grain size intersected, leading to Hall–Petch strengthening. These findings underscore the critical role of grain morphology and crystallographic texture in governing fracture resistance in LPBF-fabricated IN718.

## Figures and Tables

**Table 1:** Chemical composition of the IN718 powder (wt.%).

wt.%	Al	C	Cr	Fe	Mo	N	Nb + Ta	Ni	O	Ti	Other
Nominal	0.48	0.03	19.04	18.20	2.98	0.01	5.16	52.99	0.02	0.97	0.12
Measured	0.80	ND	20.28	18.10	2.73	ND	5.45	51.78	ND	0.86	ND

**Table 2:** PBF-LB manufacturing parameters.

Parameter	Value
Material	IN718
Hatch distance, $h_{d}$, (μm)	90
Layer thickness, *z*, (μm)	60
Laser power, Po, (W)	200
Scan speed, *v*, (mm/s)	1000
Scanning strategy, SS	R0

**Table 3:** Fracture toughness parameters for Inconel 718.

Parameter	R0-0	R0-45
*P* (MN)	0.07954	0.08711
*B* (m)	0.025	0.025
$B_{N}$ (m)	0.025	0.025
$L_{o}$ (m)	0.312	0.312
*W* (m)	0.05	0.05
*a* (m)	0.017625	0.017625
a/W	0.3525	0.3525
$f(\frac{a}{W})$	6.4331	6.4331
ν	0.33	0.33
*E* (MPa)	200,000	200,000
$E^{\prime }$ (MPa)	224,441.70	224,441.70

**Table 4:** Values of *K* and elastic component $J_{\mathrm{el}}$ for 0° and 45° orientations.

Orientation	*K* [MPa$\;\cdot \;\sqrt{m}$]	$J_{\mathrm{el}}$ [MJ/m^2^]
0°	91.5305	0.03732
45°	100.249	0.04477

**Table 5:** Plastic area ($A_{pl}$), plastic contribution to the *J*-integral ($J_{\mathrm{pl}}$), and total fracture toughness estimated via two methods ($J_{\mathrm{IC}-M1}$ and $J_{\mathrm{IC}-M2}$) for specimens oriented at 0° and 45°.

R	$A_{\mathrm{pl}}$ [MN·m]	$J_{\mathrm{pl}}$ [MJ/m^2^]	ξ [m]	$J_{\mathrm{IC}-M1}$ [MJ/m^2^]	$J_{\mathrm{IC}-M2}$ [MJ/m^2^]
0°	0.00017301	0.49977	0.0615	0.5370	0.4213
45°	0.00043639	1.26059	0.0620	1.3053	1.0714

## Data Availability

The original contributions presented in this study are included in the article. Further inquiries can be directed to the corresponding author.
